# The phagocytic state of brain myeloid cells after ischemia revealed by superresolution structured illumination microscopy

**DOI:** 10.1186/s12974-019-1401-z

**Published:** 2019-01-16

**Authors:** Stefano Fumagalli, Fabio Fiordaliso, Carlo Perego, Alessandro Corbelli, Alessandro Mariani, Massimiliano De Paola, Maria-Grazia De Simoni

**Affiliations:** 1 Department of Neuroscience, Istituto di Ricerche Farmacologiche Mario Negri IRCCS, via G. La Masa 19, 20156 Milan, Italy; 2 Department of Cardiovascular Research, Istituto di Ricerche Farmacologiche Mario Negri IRCCS, Milan, Italy; 3 Department of Environmental Health Sciences, Istituto di Ricerche Farmacologiche Mario Negri IRCCS, Milan, Italy

**Keywords:** Phagocytosis, Brain myeloid cells, Superresolution, Structured illumination microscopy, Stroke

## Abstract

**Background:**

Phagocytosis is a key function of myeloid cells and is highly involved in brain ischemic injury. It has been scarcely studied in vivo, thus preventing a deep knowledge of the processes occurring in the ischemic environment. Structured illumination microscopy (SIM) is a superresolution technique which helps study phagocytosis, a process involving the recruitment of vesicles sized below the resolution limits of standard confocal microscopy.

**Methods:**

Mice underwent permanent occlusion of the middle cerebral artery and were sacrificed at 48 h or 7 days after insult. Immunofluorescence for CD11b, myeloid cell membrane marker, and CD68, lysosomal marker was done in the ischemic area. Images were acquired using a SIM system and verified with SIM check. Lysosomal distribution was measured in the ischemic area by the gray level co-occurrence matrix (GLCM). SIM dataset was compared with transmission electron microscopy images of macrophages in the ischemic tissue at the same time points. Cultured microglia were stimulated with LPS to uptake 100 nm fluorescent beads and imaged by time-lapse SIM. GLCM was used to analyze bead distribution over the cytoplasm.

**Results:**

SIM images reached a resolution of 130 nm and passed the quality control diagnose, ruling out possible artifacts. After ischemia, GLCM applied to the CD68 images showed that myeloid cells at 48 h had higher angular second moment (ASM), inverse difference moment (IDM), and lower entropy than myeloid cells at 7 days indicating higher lysosomal clustering at 48 h. At this time point, lysosomal clustering was proximal (< 700 nm) to the cell membrane indicating active target internalization, while at 7 days, it was perinuclear, consistent with final stages of phagocytosis or autophagy. Electron microscopy images indicated a similar pattern of lysosomal distribution thus validating the SIM dataset. GLCM on time-lapse SIM from phagocytic microglia cultures revealed a temporal decrease in ASM and IDM and increase in entropy, as beads were uptaken, indicating that GLCM informs on the progression of phagocytosis.

**Conclusions:**

GLCM analysis on SIM dataset quantitatively described different phases of macrophage phagocytic behavior revealing the dynamics of lysosomal movements in the ischemic brain indicating initial active internalization vs. final digestion/autophagy.

**Electronic supplementary material:**

The online version of this article (10.1186/s12974-019-1401-z) contains supplementary material, which is available to authorized users.

## Background

**Fig. 1:**
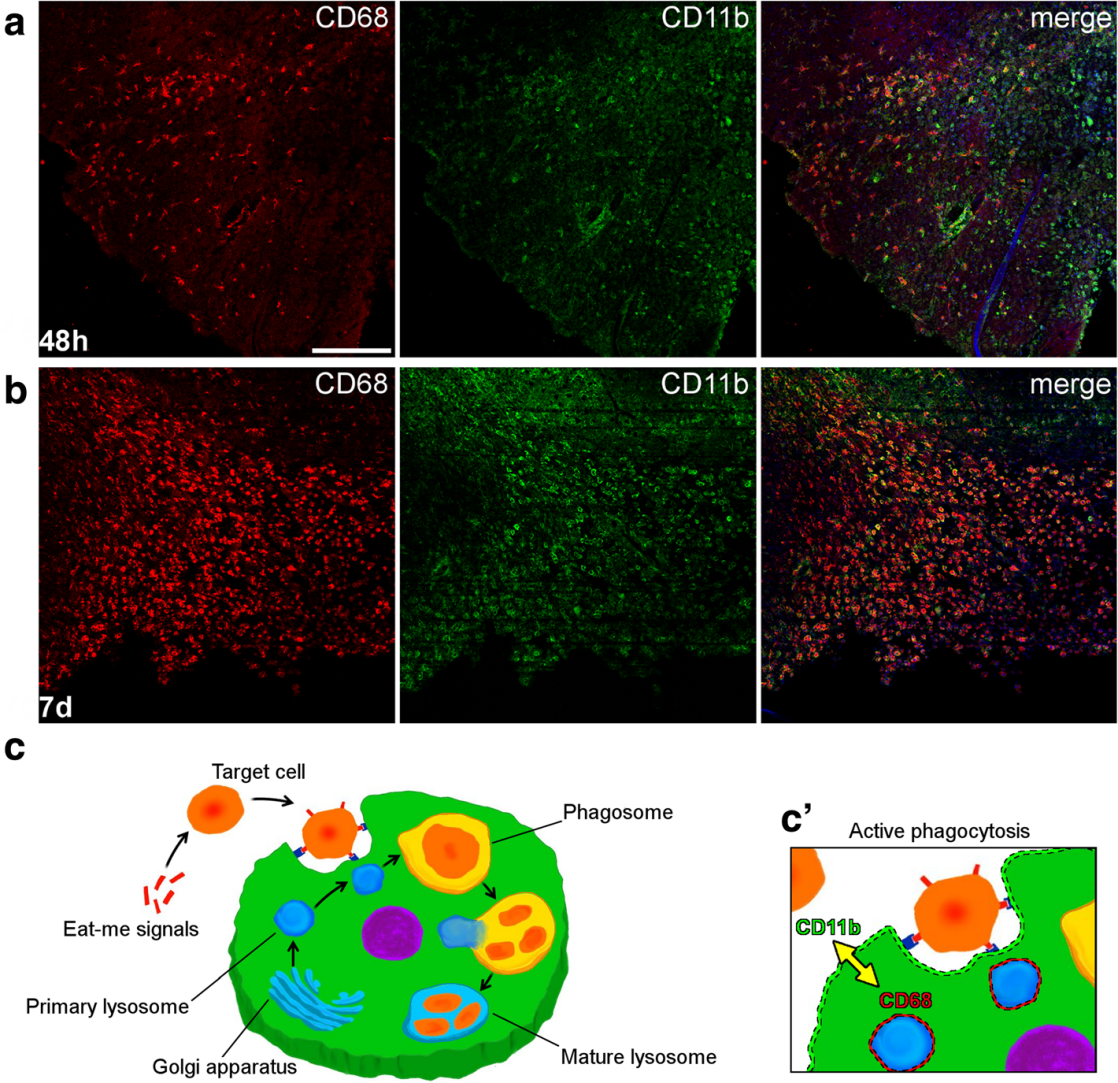
CD68-positive brain myeloid cells after permanent ischemia and simplified scheme of the phagocytic process. Confocal images of the ischemic hemisphere showing cells positive for the lysosomal marker CD68 (red) and for the myeloid cell membrane marker CD11b (green) in the ischemic area at 48 h (**a**) or 7 days (**b**) after pMCAo, the latter time showing increased density of CD68-positive cells (nuclei in blue, scale bar = 200 μm). Steps leading to phagocytosis include cell targeting by eat-me-signals, recognition by the phagocytic cell, and vesicular trafficking within the phagocyting cell. There, primary lysosomes get close to cell membrane and fuse with phagosomes. The mature lysosomes complete digestion with lysozimes, leaving residual bodies within the cytoplasm or getting back to Golgi (**c**). The proximity of lysosomes (labeled by CD68) with cell membrane (labeled by CD11b) can be exploited to measure active phagocytic internalization by confocal microscopy (**c′**)

Brain myeloid cells—microglia and macrophages—react to an acute injury like ischemia by getting access to the damaged area and by activating either a pro-inflammatory or an anti-inflammatory and regulatory behavior, whose balance impacts on injury progression [1, 2]. Key to myeloid cell reactivity is their phagocytic ability which drives either viable neuron elimination (phagoptosis, detrimental, [3]) or debris removal (protective, [4, 5]), thus contributing to ischemic pathogenesis. Phagocytosis is a finely tuned process triggered by opsonization proteins (eat-me signals) like complement active fragments C3b/iC3b recognized by the CD11b/CD18 receptor [6] or immunoglobulins recognized by Fcγ receptors [7], that cover the target and favor myeloid cell chemotaxis [8]. Myeloid cells undergo subcellular changes during phagocytosis, including plasma membrane tension increase, cytoskeletal reorganization, and vesicular trafficking [9, 10]. After increase of plasma membrane tension aimed at facilitating ingestion (first phase), lysosomes are sent to the cell membrane where they fuse with phagosomes creating phagolysosomes (second phase, [10]). The second phase of phagocytosis is completed by lysozime-induced digestion and lysosome recycling involving Golgi and endoplasmatic reticulum compartments (Fig. 1).

At present, there are few tools to reliably study phagocytosis and most of them are based on in vitro approaches [11]. However, myeloid cells exploit their full potential in vivo, since the damaged microenvironment drives their activation and phenotypical changes [2, 12]. Imaging techniques may be used to visualize phagocytosis by labeling for CD68 (macrosialin), a member of the lysosome-associated membrane glycoprotein (LAMP) family [13, 14]. The CD68-associated signal can be visualized and quantified by different optical imaging approaches [1, 15, 16]. However, the optical techniques widely employed lack sufficient resolution to properly resolve small structures like lysosomes (as small as 100 nm in diameter) and might yield biased results. As such, superresolution microscopy offers a tool to overcome the limits of conventional optical microscopy.

The advent of techniques of superresolved optical microscopy has opened new possibilities of imaging by extending the resolution limits to a nanometric scale [17–19]. There is wide literature documenting the effective resolution achievable by these techniques by proof-of-concept protocols. However, the use of superresolution microscopy in practical experimental settings is often limited by factors hampering the effective resolution, like tissue scattering, sample preparation, and/or microscope set up [20]. As a consequence of these limitations, superresolution microscopy might yield image artifacts leading to misinterpretation of biological information [21]. For this reason, superresolution protocols need thorough quality validation prior to use in experimental settings. Here, we focused on neuroinflammation, a research field which may benefit from high-quality superresolved imaging since inflammatory cells contain small-sized vesicles underpinning their functions. We have applied and validated superresolution by structured illumination microscopy (SIM) to analyze brain myeloid cells after acute brain injury.

Lysosomes are the major digestive compartment of macrophages and represent the final step of the degradative endocytic pathway [22]. During the immune response, lysosomes continuously traffic from the trans-Golgi network to fuse with phagosome or recycle lysosomal components [22]. Our knowledge of phagocytosis mainly comes from in vitro studies and the steps of phagocytosis in brain myeloid cells within the ischemic territory are still unknown. Of note, brain immune mechanisms present key differences compared to what happens systemically, given the presence of the blood-brain barrier and of an incomplete lymphatic bed, overall potentially impacting on the phases of immune cell maturation and functions.

Here, we present a new approach based on SIM—providing superresolved optical imaging—to measure phagocytosis in brain histological preparations. The protocol uses a mouse model of brain ischemia induced by permanent middle cerebral artery occlusion (pMCAO), with analysis done at two times after ischemia, representing different temporal phases of phagocytosis. We also provide a full validation of the method by assessing (1) the quality of the superresolved acquisition dataset by software-based image diagnose, (2) the occurrence of image artifacts by comparing SIM images with transmission electron microscopy (TEM) images, and (3) the functional meaning of SIM image analysis by time-lapse imaging of phagocytosis in live microglia cultures.

## Methods

### Animals

We used male C57BL6/J mice (20–25 g, 10-week-old, Envigo Italy), housed in a specific pathogen free vivarium at a constant temperature (21 ± 1 °C) with a 12 h light–dark cycle and free access to food and water. The Istituto di Ricerche Farmacologiche Mario Negri IRCCS adheres to the principles set out in the following laws, regulations, and policies governing the care and use of laboratory animals: Italian Governing Law (D.lgs 26/2014; Authorisation 19/2008-A issued March 6, 2008 by Ministry of Health); Mario Negri Institutional Regulations and Policies providing internal authorisation for conducting animal experiments (Quality Management System Certificate—UNI EN ISO 9001:2008—Reg. No 6121); the NIH Guide for the Care and Use of Laboratory Animals (2011 edition); and EU directives and guidelines (EEC Council Directive 2010/63/UE). The statement of compliance (assurance) with the Public Health Service (PHS) Policy on Human Care and Use of Laboratory Animals was reviewed recently (9/9/2014) and will expire on September 30, 2019 (Animal Welfare Assurance #A5023-01). All procedures regarding the study design, animal experiments, statistical analysis, and data reporting fulfill the criteria of the (Additional file 1) ARRIVE (Animal Research: Reporting of In Vivo Experiments) guidelines (https://www.nc3rs.org.uk/arrive-guidelines).

### Experimental design, randomization, and blinding

Mice were randomly allocated to experimental groups (48 h vs. 7 days post-ischemia) and assigned across cages and experimental days. To minimize variability, all surgeries were done by the same investigator. Subsequent evaluations were done by blinded investigators.

### Focal cerebral ischemia

Permanent ischemia was done by permanent middle cerebral artery occlusion (pMCAO, [1, 23]. Anesthesia was induced by 3% isoflurane inhalation in a N_2_O/O_2_ (70/30%) mixture and maintained by 1% to 1.5% isoflurane inhalation in a N_2_O/O_2_ (70/30%) mixture. A vertical midline incision was made between the right orbit and tragus. The temporal muscle was excised, and the right middle cerebral artery (MCA) was exposed through a small burr hole in the left temporal bone. The dura mater was cut with a fine needle, and the MCA permanently occluded by electrocoagulation just proximal to the origin of the olfactory branch. Permanent occlusion of the MCA was confirmed visually before closing the wound with sutures as previously described [23]. Intraoperative rectal temperature was kept at 37.0 ± 0.5 °C using a heating pad (LSI Letica). Mortality was 0%. At sacrifice, mice were transcardially perfused with 20 mL of PBS, 0.1 mol/liter, pH 7.4, followed by 50 mL of chilled paraformaldehyde (4%) in PBS. After carefully removing the brains from the skull, they were transferred to 30% sucrose in PBS at 4 °C overnight for cryoprotection. The brains were then rapidly frozen by immersion in isopentane at − 45 °C for 3 min and stored at − 70 °C until use.

### Microglia cell cultures

Primary cultures of microglia were obtained from the spinal cord of 13-day-old C57 BL6/J mouse embryos as previously described [24]. Briefly, ventral horns were dissected from spinal cords, exposed to DNAse and trypsin, and centrifuged through a bovine serum albumin gradient. Cells obtained at this step were a mixed neuron/glia population and underwent centrifugation through a 6% iodixanol (OptiPrep™; Sigma Aldrich S.r.L.) cushion to separate neurons from glial cells. The glial fraction was plated at a density of 25,000 cells/cm^2^ into 75 cm^2^ flasks, previously pre-coated with poly-l-lysine. Isolated microglia were obtained from flasks containing confluent mixed glial cultures after overnight orbital shaking at 275 rpm in incubators. The supernatants (containing detached microglia) were collected and seeded at a density of 60,000 cells/cm^2^ into microslides (8-well μSlides; Ibidi GmbH) for time-lapse analysis. Primary microglia were activated by treatment with 1 μg/mL lypopolysaccharide (LPS) (from *Escherichia coli* 0111:B4; Sigma Aldrich S.r.L.) on the fifth-sixth day in vitro (5–6 DIV) for 18 h [24]. Cultures maintained with normal medium were the control condition. After LPS treatment, microglial cells were stained with far red fluorescent dye (CellTrace™ Far Red Cell Proliferation Kit; Thermo Fisher Scientific Inc.) to allow cell tracking in live-cell imaging experiments. Green fluorescent 100 nm beads (Alexa 488 conjugated beads used at dilution 1:10000, Thermo Fisher Scientific Inc.) were then added to cell cultures 20 min before the time-lapse acquisition. After live-imaging acquisition, cells were fixed in 4% formaldehyde solution, permeabilized with 0.3% Triton X-100 (Sigma Aldrich S.r.L.), and stained with anti-CD68 primary antibody (1:200; Serotec, Kidlington, UK) followed by Alexa 546 anti-rat secondary antibody (1:500, Invitrogen, Carlsbad, CA).

### Optical imaging

#### Tissue preparation

Brains were collected after transcardiac perfusion with 30 mL PBS 0.1 M and 60 mL PAF 4% and frozen in isopenthane, 3 min at − 45 °C. Frozen brains were serially cut at the cryostate into 20 μm coronal sections. Before immunofluorescence, tissues were post-fixated with acetone followed by ethanol 100%, 20 s each. Sections were then washed three times with PBS 0.01 M. Immunofluorescence was performed according to the previously described method [15]. Primary antibodies used were anti-mouse CD11b (1:30000, BioRad) and anti-mouse CD68 (1:200, Serotec, Kidlington, UK). Secondary antibodies used were Alexa 546 anti-rat (1:500, Invitrogen, Carlsbad, CA) and biotinylated anti rat (1:200, Vector Laboratories, Burlingame, CA), this latter followed by fluorescent signal coupling with streptavidine TSA amplification kit (cyanine 5, Perkin Elmer, MA, USA). Appropriate negative controls were run without the primary antibodies. None of the immunofluorescence reactions gave unspecific fluorescent signal in the negative controls.

#### Image acquisition—confocal

Confocal microscopy was done on a Nikon A1 confocal scan unit with a 20 × 0.5 numerical aperture (NA) or a 100 × 1.49 NA oil immersion objective (common to the SIM system), managed by NIS elements software. Tissues were imaged at laser excitation of 405 (for nuclei), 561 (for CD68), and 640 nm (for CD11b) with a sequential scanning mode to avoid bleed-through effects. The whole hemisphere was acquired with the × 20 objective, using the large field acquisition command with 15% image overlapping to allow stitching. Each image had a pixel size of 1.2 μm and was acquired over a 15 μm *z*-axis (step size = 2.4 μm). The acquisition of the field of view of the SIM images was done with the × 100 objective zoomed to reach a 0.03 μm pixel size (same of SIM). Images were managed with ImageJ and finally elaborated with GIMP.

#### Image acquisition—SIM

Structured illumination microscopy (SIM) on brain sections was done on a Nikon SIM system with a × 100 1.49 NA oil immersion objective, managed by NIS elements software. Tissues were imaged at laser excitation of 405 (for nuclei), 561 (for CD68), and 640 nm (for CD11b) with a 3D-SIM acquisition protocol. Sixteen-bit images sized 1024 × 1024 pixels with a single pixel of 0.030 μm were acquired in a gray level range of 0–16,000 to exploit the linear range of the camera (iXon ultra DU-897 U, Andor) and to avoid saturation. Five cells per mouse were selected and acquired in the ischemic core. Raw and reconstructed images were validated with the SIMcheck plugin of ImageJ [25]. SIM on cultured microglia was done with the same system using a × 60 1.27 numerical aperture water immersion objective, being the cells in a aqueous medium. Cells were kept at 37 °C, 5% CO_2_ and 90% humidity, all feedback controlled, in a microscope incubator (H-301 unit, Okolab, managed by Oko-touch controller). The objective was kept at the same temperature of the culture by a heated sleeve connected to the incubator controller. The fluorescent beads were imaged at laser excitation of 488 nm with a 3D-SIM acquisition protocol. Fourteen-bit images sized 1024 × 1024 pixels with a single pixel of 0.036 μm were acquired in a gray level range of 0–4000 to exploit the linear range of the camera at 14-bit and to avoid saturation. Time lapse was done over 170' with a 30′ time frame interval, with manual refocusing before each scan as the cells slightly displaced. The fluorescent marker labeling the cells’ cytoplasm was acquired at 640 nm using the wide-field modality just after the SIM acquisition of the beads. Images were finally elaborated with GIMP.

#### Image analysis

SIM images were converted to 8-bit for the gray level co-occurrence matrix (GLCM) analysis. After background normalization, the cell outline was manually traced over the CD11b membrane signal and used like the region of interest (ROI). GLCM was done on the CD68 signal within the ROI with the ImageJ plugin ‘GLCM analysis’ (using step size = 1 pixel, step direction = 0°). Cells were segmentated based on their outline and analyzed for morphology as described in [26]. For the analysis of the CD68 signal at different distances from the membrane, an ImageJ algorithm was created to draw 100-, 300-, 500-, and 700-nm-thick boundaries from the CD11b outline to the cytoplasm. The integrated density of the CD68 signal was calculated in the total ROI and within each of the boundaries. The data were plotted as fraction of the total integrated density. To quantify the uptake of fluorescent beads by cultured microglia, the beads were segmentated to obtain a binary image. The number of segmentated beads inside the cell cytoplasm (manually traced) and in a 700-nm-thick boundary was calculated. ImageJ was used for this analysis. GLCM on time-lapse images was done using step size = 1 pixel, step direction = 0°.

### TEM

#### Tissue preparation

Mice were deeply anesthetized and perfused through the ascending aorta with phosphate-buffered saline (PBS, 0.1 M; pH 7.4) followed by 2% paraformaldehyde (PFA) and 2.5% glutaraldehyde in PBS. The area of hypoxic damage was excised and reduced and post-fixed with 4% paraformaldehyde (PFA) and 2% (wt/vol.) glutaraldehyde in phosphate buffer 0.12 mol/l pH 7.4 overnight at 4 °C, followed by incubation at room temperature for 2 h in 1% (wt/vol.) OsO_4_. After dehydration in a graded series of ethanol preparations, tissue samples were cleared in propylene oxide, embedded in epoxy medium (Epoxy Embedding Medium kit; Sigma-Aldrich, St. Louis, MO 63103 USA), and polymerized at 60 °C for 72 h. From each sample, one semi-thin (1 μm) section was cut with a Leica EM UC6 ultramicrotome (Leica Microsystems, Vienna, Austria), stained with Toluidine Blue and mounted on glass slides to identify the ischemic area. Ultra-thin (60 nm thick) sections of areas of interest were then obtained, counterstained with uranyl acetate and lead citrate, and examined with an energy filter transmission electron microscope (Libra120, Carl Zeiss NTS GmbH, Oberkochen, Germany).

#### Image acquisition

The ipsilateral side to the lesion and its contralateral not damaged area from two mice from each experimental groups (48 h vs. 7 days post-ischemia) were thoroughly observed to define the ischemic area to be acquired (Additional file 2: Figure S1). Images of 25 macrophages for each mouse were acquired by the iTem software (Olympus Soft Imaging Solutions, Germany) coupled with a yttrium aluminum garnet (YAG) scintillator slow-scan charge-coupled device (CCD) camera (Sharp eye, TRS, Moorenweis, Germany).

#### Image analysis

A ROI was delineated by a manual tracing of the cell membrane. Then an ImageJ algorithm was created to draw a 700-nm-thick boundary from the membrane outline to the cytoplasm. Lysosomes were segmented based on a color threshold and their number within the total ROI or within the 700-nm-thick boundary was calculated. The data are expressed as fraction of lysosomes lying in the 700 nm boundary.

### Statistical analysis

All data are presented as mean and standard deviation (sd). Statistical power (1-β) was assessed as post-hoc analysis by means of G*Power [27]. GraphPad Prism (GraphPad Software Inc., San Diego, CA, USA, version 7.0) was used for group comparisons. Groups were compared using *t* test or two-way ANOVA followed by an appropriate post hoc test. *p* values lower than 0.05 were considered statistically significant. A detailed description of the test used is provided in the figure legends.

## Results

### CD68-positive cell distribution in the ischemic brain and scheme of the phagocytic process

After pMCAo, CD68-positive myeloid cells were recruited to the ischemic core area (Fig. 1(a, b)). In line with previous data [1], their density was higher at 7 days than 48 h after pMCAo. CD68 (macrosialin) is a member of the lysosome-associated membrane glycoprotein (LAMP) family, whose localization and predominance in phagocytic macrophages implicates CD68 in phagocytosis [13, 14]. Phagocytosis is triggered by eat-me-signal molecules that tag the target cell, which is subsequently recognized by a phagocytic cell. A scheme of the phagocytic process is depicted in Fig. 1) Proximity of lysosomes (labeled by CD68) to myeloid cell membrane (labeled by CD11b, an integrin receptor) occurs during the active internalization phases of phagocytosis (Fig. 1(c′)).

### Confocal microscopy vs. SIM and validation of SIM images

**Fig. 2:**
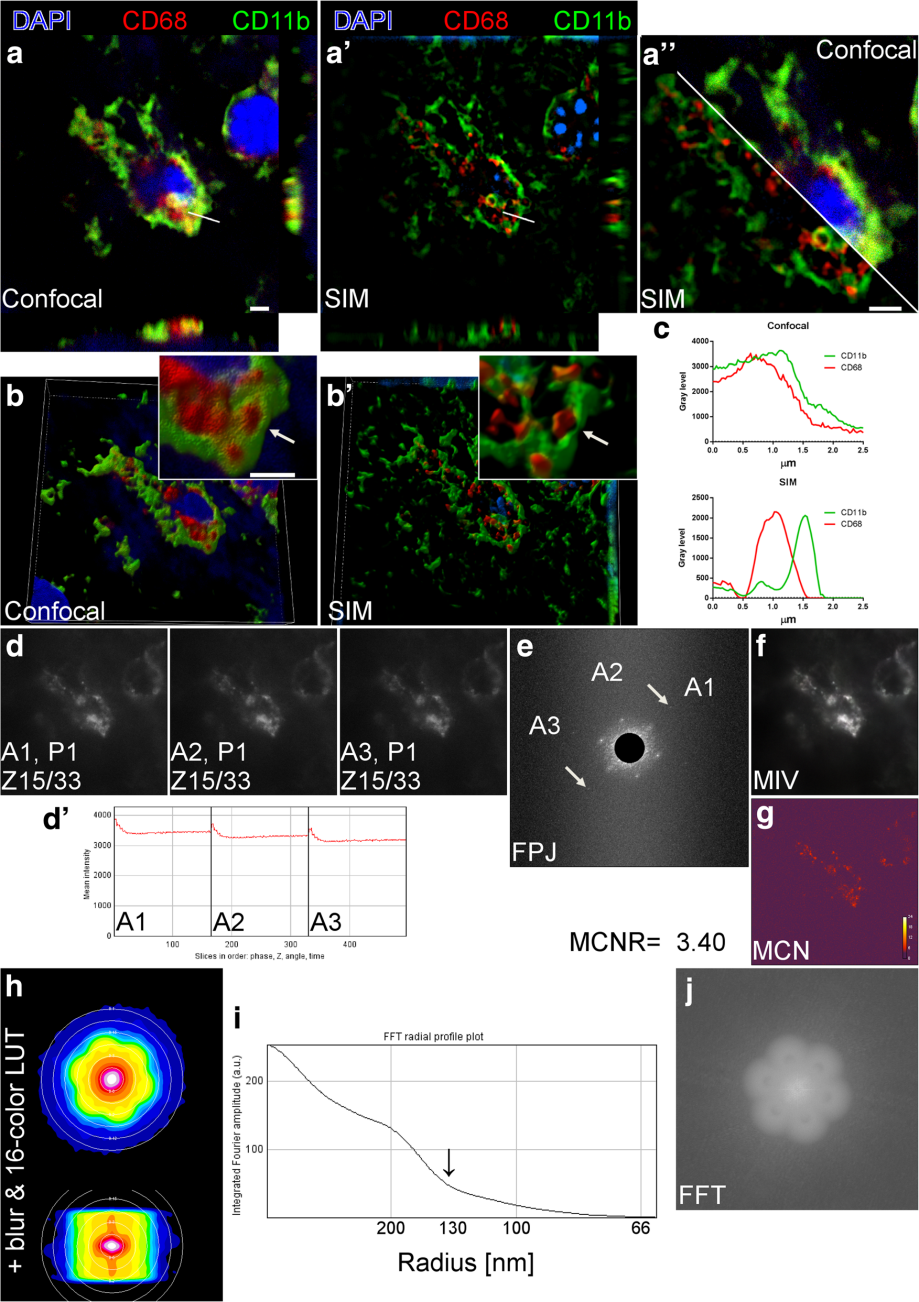
Confocal microscopy vs. SIM and CD68 SIM dataset validation by image diagnosis. Planar *xy* view with *xz* (below) and *yz* (right) projections of CD11b (green) and CD68 (red) showing merged signal (yellow) by confocal microscopy (**a**), or signal proximity with no colocalization by SIM (**a′**). Image overimposition details the different resolutions achieved by confocal microscopy vs. SIM (**a′′**). Three-dimensional view of the same cell by confocal (**b**) and SIM (**b′**). The arrow in (**b**) show a lysosome (CD68) entirely surrounded by cell membrane (CD11b) in confocal microscopy. SIM yields enough resolution to visualize lysosome proximity to cell mebrane with no fusion (**b′**, arrow). Scale bars = 2 μm. Plot profiles over the lines in a and a′ confirm that SIM, but not confocal microscopy, allows to visualize close, non-overlapping CD11b and CD68 signals (**c**). SIMcheck output for 3D-SIM raw dataset of CD68 showing the three illumination angles at the first phase (P1) and at the 15th focal plane (Z15/33,). **d** The channel intensity profile shows limited intensity variation over phases (**d′**). Raw Fourier projection (FPJ) showing points of high-frequency information for each angle from first- (inner spots) to second-(outer spots, arrows for angle 1) order stripes (**e**). Second-order spots in angle 2 are less intense. Motion and illumination variation (MIV) based on phase-averaged and intensity-normalized images for each angle showing a gray-white merge output, thus indicating motion stability and evennes of the illumination (**f**). Modulation contrast-to-noise ratio (MCN) showing the heatmap of local contrast which is slightly unsatisfactory (< 4, **g**). SIMcheck output for 3D-SIM reconstructed dataset of CD68 showing the ‘flower’ pattern in 16-color-coded image for the *xy* plane and the *xz* projection (**h**). The inflection point in the radial profile plot indicates approximate effective resolution achieved in the reconstructed data (≈130 nm, arrow, **i**). Fast Fourier transform (FFT) showing the extended focus image of the ‘flower’ pattern of the reconstructed image (**j**)

Confocal microscopy of myeloid cells labeled for CD68 and CD11b showed partial signal co-localization (Fig. 2(a)), while SIM allowed to visualize a gap between the two signals (Fig. 2(a′, a′′)). The three-dimensional views of confocal (Fig. 2(b)) or SIM (Fig. 2(b′)) images provided better resolved details reached by SIM, as supported by the plot profiles shown in Fig. 2(c). To validate the SIM images, test microscope calibration, and rule out potential artifacts, SIMcheck was applied to raw and reconstructed SIM datasets for CD68 signal. As for the raw images, the channel intensity profiles from the three grating angles (Fig. 2(d)) showed little variation throughout the different phases (Fig. 2(d′)). The raw Fourier projection (FPJ) showed good second-order spots for angle 1 and 3, while pale spots for angle 2 (Fig. 2(e)). Motion and illumination variation (MIV) indicated satisfactory motion stability and evennes of the illumination since the predominant color was gray-white (Fig. 2(f)). Modulation contrast-to-noise ratio (MCN) showed a poor contrast (< 4, Fig. 2(g)). Overall the satisfactory tests (even intensity profiles, FPJ and MIV) indicated that the system was well calibrated, the acquisition parameters were properly set, and the sample was correctly prepared. However, the poor contrast shown by MCN indicates a strong background noise, likely due to the damaged tissue in the ischemic core. The analysis of the reconstructed image showed that the light was collected according to the typical ‘flower’ pattern of SIM (Fig. 2(h)). The inflection point in the radial profile plot allowed to known the effective resolution achieved after data reconstruction, which was ≈130 nm, thus close to the theoretical 100 nm SIM limit (Fig. 2(i)). The fast Fourier transform (FFT) over the extended focus image confirmed the ‘flower’ pattern of the reconstructed image (Fig. 2(j)). The same analysis was done for the nuclei and the CD11b signals, shown in Additional file 2: Figure S2 and Figure S3 respectively.

### Quantification of lysosome distribution with gray level co-occurrence matrix

**Fig. 3:**
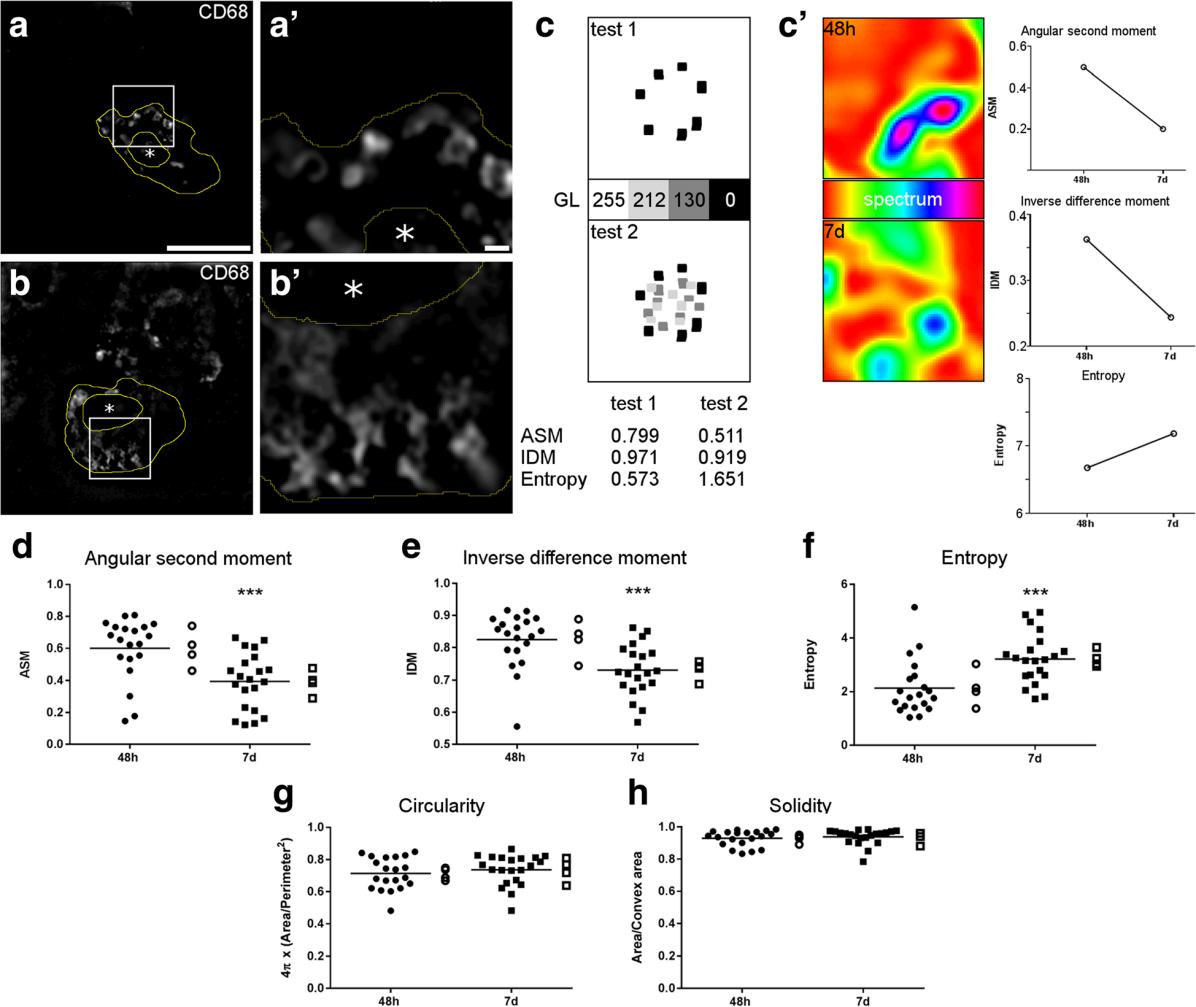
Gray-level co-occurrence matrix (GLCM) for the quantification of lysosome intracellular distribution. Representative images of the CD68 labeling showing clustered lysosomes at 48 h (**a, a**′) vs. spread lysosomes at 7 days (**b, b′**) after pMCAo (yellow outline obtained tracing over CD11b, asterisks indicate nuclei). Scale bars = 10 μm (**a**) and 1 μm (**a′**). Testing images on four different gray levels (GL) representing clusters of pixels with same GL (test 1) vs. pixels with different GL (test 2, **c**). With gray-level co-occurrence matrix analysis, test 1 showed higher angular second moment (ASM) and inverse difference moment (IDM) and lower entropy than test 2. Testing images from 48 h and 7 days pMCAo mice showing the CD68-positive pixels in a spectrum modality (**c′**). GLCM showed higher pixel homogeneity at 48 h, when clustered pixels were found (**c′**, right graphs). GLCM on CD68-positive pixels showing higher ASM and IDM (**d**, **e**) and lower entropy (**f**) at 48 h than 7 days. Morphological parameters like circularity (**g**) and solidity (**h**) did not differ between time points, thus ruling out any contribution of cell morphology to pixel distribution analysis. Individual cell values with line at mean (*n* = 20 cells, full points) and mean values per mouse (*n* = 4 mice, empty points). Normal distribution by Kolmorgov-Smirnov analysis, *t* test, ***p < 0.001

CD68-labeled lysosomes distributed differently in the myeloid cell cytoplasm depending on the time point after pMCAo. At 48 h, lysosomes appeared to be clustered proximal to the CD11b-labeled cell membrane (Fig. 3(a, a′)), while at 7 days, they were more spread over the cytoplasm (Fig. 3(b, b′)). To have a quantitative evaluation of lysosomal distribution, we applied gray level co-occurrence matrix (GLCM) to CD68 images. GLCM measures pixel homogeneity providing angular second moment (ASM), inverse difference moment (IDM), and entropy as output data. We used GLCM with two sample images, one with cluster of black pixels (0 gray level, GL) on a white background (255 GL, test 1) and the other with additional clusters of pixels with 212 and 130 GL (test 2, Fig. 3c). Test 1 had higher ASM (0.799) and IDM (0.971) and lower entropy (0.573) than test 2 (0.511, 0.919 and 1.651), all data indicating higher pixel homogeneity in test 1. Indeed in test 1, a given pixel is likely to have neighbor pixels with same GL, either black (0 GL) or white (255 GL), resulting in high homogeneity. On the contrary, in test 2, a given pixel might have neighbor pixels with different GL (including 212 or 130 GL). Similar results were obatined using test images of CD68 showing different positive pixel clustering (Fig. 3(c′)). Using GLCM on the CD68 images, myeloid cells at 48 h had higher ASM (0.60 ± 0.19, mean ± sd), IDM (0.82 ± 0.09), and lower entropy (2.14 ± 1.02) than myeloid cells at 7 days (ASM: 0.39 ± 0.18, IDM: 0.73 ± 0.08, entropy: 3.22 ± 0.92) after pMCAo (Fig. 3(d–f)). To rule out the bias due to cell morphology, potentially affecting the GLCM analysis, we measured two shape descriptors like circularity and solidity [26]. Myeloid cells at the two analyzed times did not differ for circularity (Fig. 3(g)) or solidity (Fig. 3(h)).

### CD68 cluster analysis at different distances from the cell membrane

**Fig. 4:**
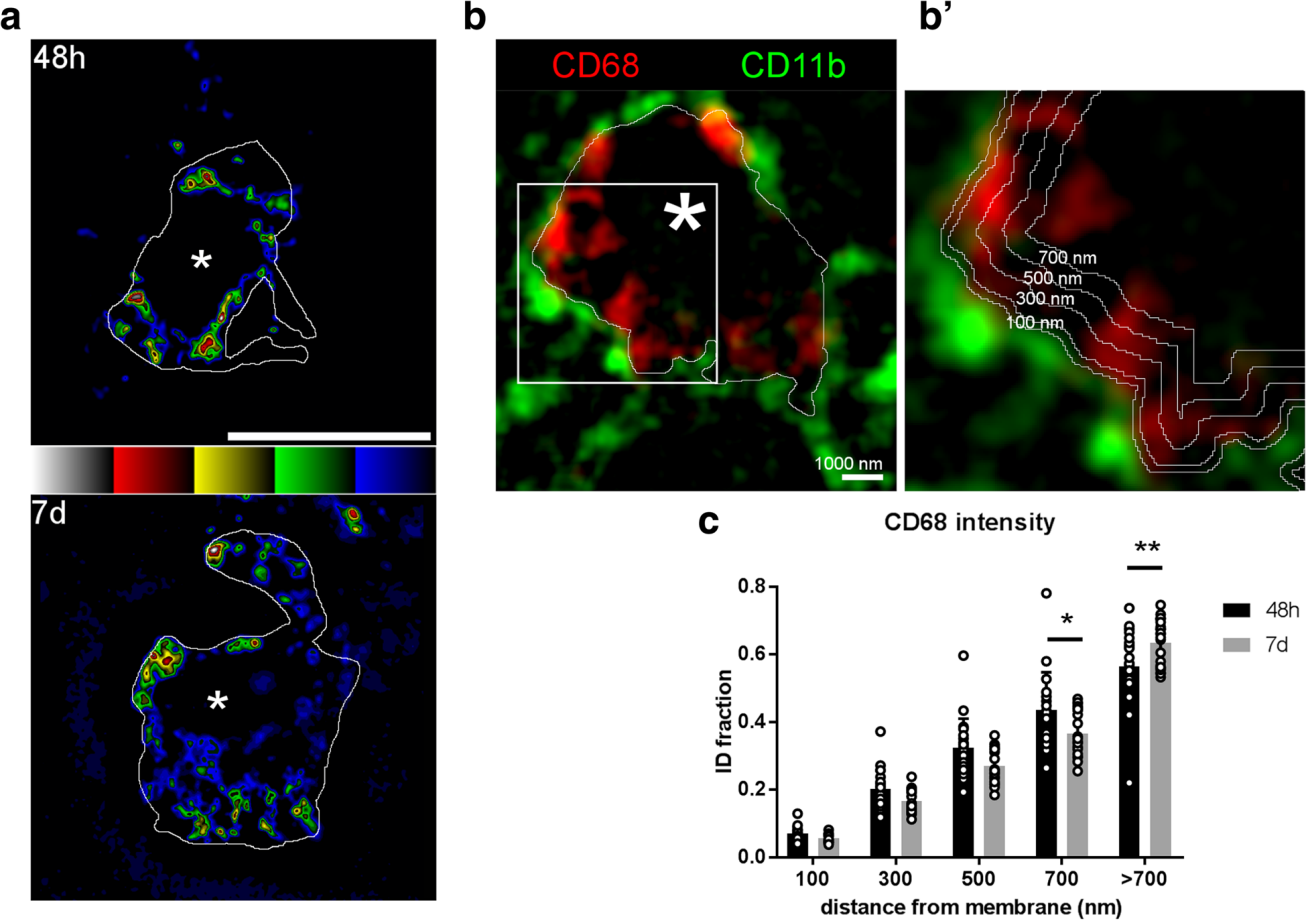
CD68 pixel clusters move away from the cell membrane from 48 h to 7 days after injury. GLCM data are consistent with increased lysosome clustering at 48 h than 7 days, as detailed by five-color-coded ramp images (**a**) showing spreading of low GL values (blue-green) at 7 days (white outline representing CD11b inner boundary, asterisks indicate nuclei, scale bar = 10 μm). After manual tracing of the inner CD11b boundary (white outline, **b**, scale bar = 1000 nm), CD68 signal intensity was analyzed at different distances (100, 300, 500, 700, and > 700 nm from the traced boundary, **b′**). CD68 integrated density (ID) fraction was higher within 700 nm and conversely lower at > 700 nm from the membrane inner interface at 48 h than at 7 days (**c**). Individual cell values with bars at mean, *n* = 20 cells, normal distribution by Kolmorgov-Smirnov analysis, two-way ANOVA *F*(4, 152) = 5.826, followed by Sidak’s post-hoc test,***p < 0.01

The five-color-coded ramp images of the SIM acquisitions of CD68 staining showed groups of clustered pixels with similar GL both at 48 h and 7 days after pMCAo (Fig. 4(a)). At 48 h, these clusters appeared to be mainly proximal to the cell membrane which was outlined tracing the CD11b signal. At 7 days, some half-tone pixels were also found in cytoplasmatic areas far from the membrane boundary. To quantify the CD68 signal distribution relative to cell membrane boundary, multiple regions of interest were obtained at increasing distances (100, 300, 500, and 700 nm) from the cell membrane edge (Fig. 4(b, b′)). The integrated density fraction of CD68 was higher within 700 nm from membrane in 48 h than 7 days post-ischemic myeloid cells (Fig. 4(c)). Conversely, at distances > 700 nm from the membrane boundary, CD68 integrated density fraction was lower 48 h than 7 days post-ischemic myeloid cells (Fig. 4(c)), indicating that from 48 h to 7 days, lysosomes progressively move away from the cell membrane.

### Validation of the SIM dataset with TEM

**Fig. 5:**
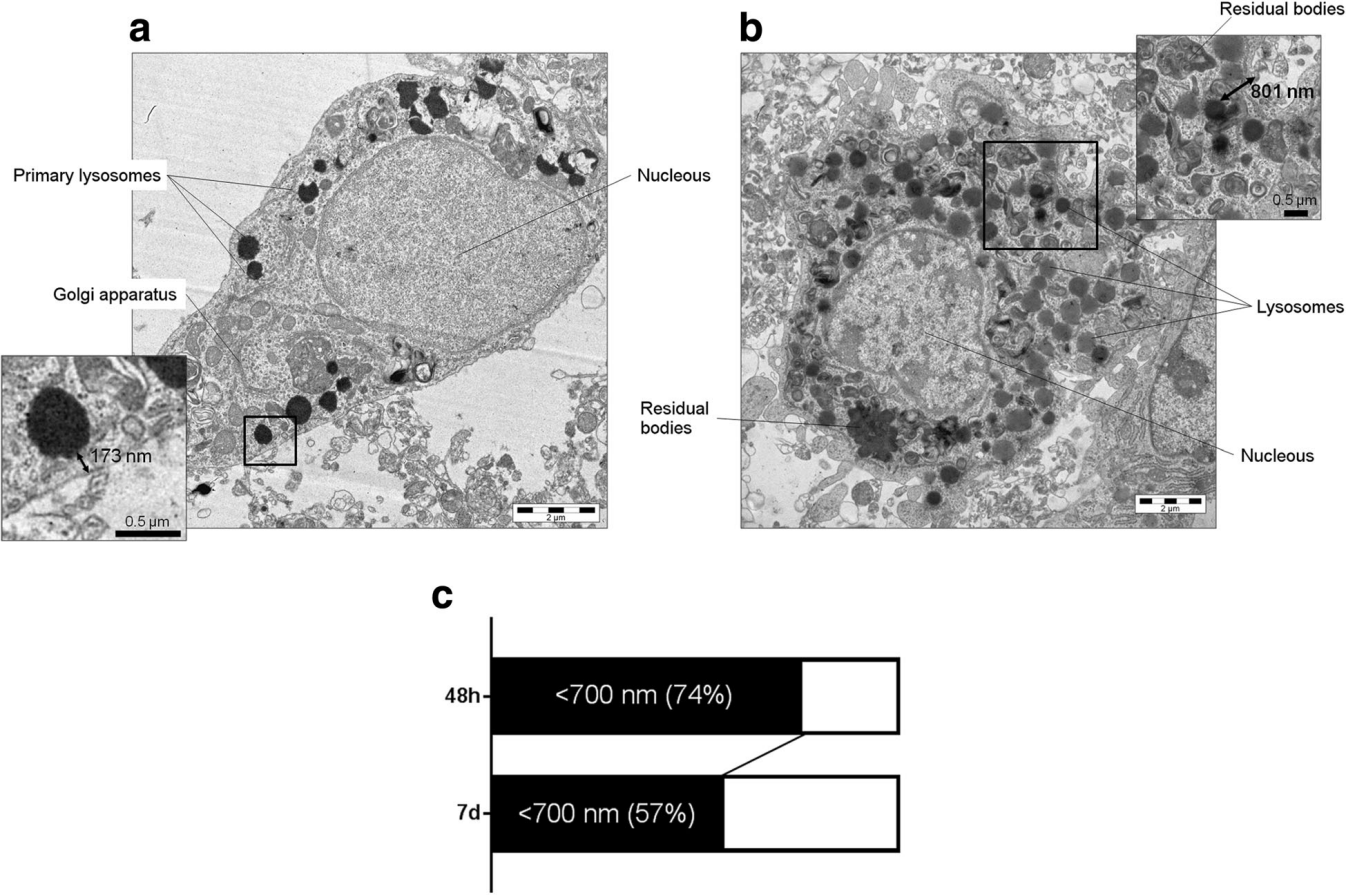
Transmission electron microscopy (TEM) images of macrophages at 48 h and 7 days after pMCAo. TEM showing a macrophage in the ischemic area at 48 h (**a**) or 7 days (**b**) after pMCAo. Scale bar = 2 μm. The macrophage at 48 h had primary lysosomes clustered close to the cell membrane (see the magnificated insert, a, scale bar = 0.5 μm), while at 7 days it was over-loaded of lysosomes at different maturation stages positioned further from cell membrane (see the magnificated insert, **b**, scale bar = 0.5 μm). At 48 h, 74% of total lysosomes were at < 700 nm from the cell membrane, while at 7 days, only the 57% of lysosomes were at this distance (**c**)

A second group of pMCAo mice was prepared and sacrificed at 48 h or 7 days after ischemia for TEM imaging. At 48 h, myeloid cells in the ischemic territory showed primary lysosomes located close to the cell membrane (Fig. 5a, the insert shows a lysosome at 173 nm from the membrane). At 7 days, myeloid cells had a dramatic increase in the number of lysosomes at different maturation stages and we could also observe phagolysosomes (produced by the fusion of lysosomes with phagosomes) and residual bodies (Fig. 5b). Lysosomes could be found also far from the membrane (see insert in Fig. 5b showing a lysosome at 801 nm from membrane) and inclusion bodies were clearly detectable. Quantification of the number of lysosomes in TEM images showed that at 48 h, the 74% were at < 700 nm from membrane, while at 7 days, only 57% (Fig. 5c), therefore confirming the SIM dataset analysis on lysosomal distribution.

### Time-lapse SIM of cultured live microglia

**Fig. 6:**
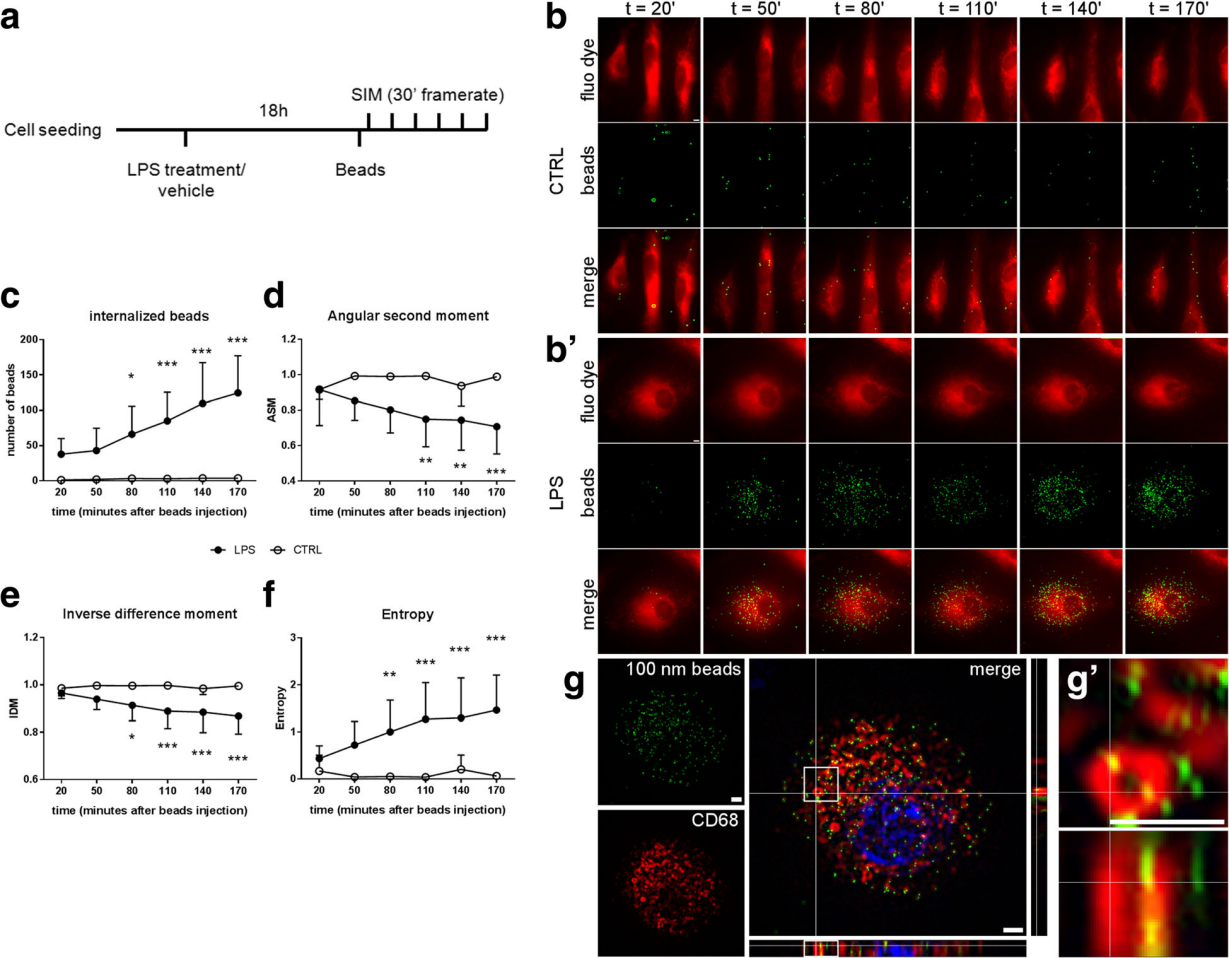
Time-lapse SIM of phagocytosis in cultured microglia. Cultured microglia were treated for 18 h with LPS to induce activation or with vehicle as control (CTRL) and exposed to fluorescent 100 nm beads. SIM started 20 min after bead exposure and was repeated with a 30-min time frame (**a**). Representative SIM images showing cultured unstimulated microglia (CTRL, **b**) or LPS-activated microglia (LPS, **b′**) over the time points. Scale bars = 2 μm. Beads were uptaken only by LPS-activated microglia as confirmed by quantification of the number of internalized beads (**c**). GLCM analysis showed time-dependent decrease of angular second moment (ASM) and inverse difference moment (IDM, **d**, **e**) in phagocytic microglia, indicative of decreased pixel homogeneity. Conversely, entropy increased over time with phagocytosis (**e**). Mean ± sd, *n* = 9. Two-way ANOVA for repeated measures (treatment effect had *p* < 0.0001 in **c**, **e**, and **f** and *p* = 0.0005 in d) followed by Dunnett’s post-hoc test, *p < 0.05, **p < 0.01, ***p < 0.001 vs. LPS *t* = 20′. Immunofluorescence on LPS microglia fixed after the final time point and SIM revealed that beads (green) were mostly uptaken by lysosomes (CD68, red, **g**) as further supported by the magnified field with the xz axis (**g′**). Scale bars = 2 μm

In order to check whether GLCM on SIM images could consistently inform on the progress of phagocytosis, cultured microglia were stimulated with LPS to promote fluorescent bead phagocytosis, and then imaged with time-lapse SIM (Fig. 6(a)). After 18 h, LPS treatment increased microglia phagocytosis of fluorescent 100 nm beads, showing a time-dependent uptake that was not observed in untreated (CTRL) microglia (Fig. 6(b, b′, c)). GLCM on SIM images revealed a temporal decrease in ASM and IDM (Fig. 6(d, e)) and increase in entropy (Fig. 6(f)), as beads were uptaken. Indeed, LPS-stimulated microglia internalized 37.89 ± 22.23 (mean ± sd) beads after 20′ exposure (first time point) vs. 124.56 ± 52.75 after 170′ (final time point). During this time frame, ASM decreased from 0.92 ± 0.06 to 0.71 ± 0.16 and IDM from 0.97 ± 0.02 to 0.87 ± 0.08, while entropy increased from 0.44 ± 0.27 to 1.46 ± 0.75. Immunofluorescence on fixed cells after the final time point showed that beads were mostly associated with CD68 positive lysosomes in LPS-stimulated microglia (Fig. 6(g, g′)). LPS-stimulated microglia with no bead exposure showed few lysosomes, thus indicating that microglia activated the phagocytic pathway only in the presence of the beads (Additional file 2: Figure S4). SIM on in vitro cultures effectively provided better resolution than confocal microscopy allowing satisfactory bead detection (Additional file 2: Figure S4).

## Discussion

This study provides an original method to analyze the phagocytic behavior of immune cells in a damaged environment based on superresolution SIM imaging. Superresolved imaging approaches widened the application of optical microscopy enabling imaging at a nanometric scale. Superresolution is regarded as a major advance to study inflammatory cells, whose function involves vesicle trafficking and plasma membrane modifications hardly detectable by diffraction-limited microscopy. Methods to study T cell membrane protein assemblies sized ≈200 nm [28] or the content of platelet granules sized 150–400 nm [29] or vesicle fusing with membrane of neuroendrocrine cells [30] have been developed based on superresolution. Lysosomes are vesicles sized 100–200 nm participating to phagocytosis. We imaged lysosomes by SIM providing higher details than confocal microscopy. Indeed, while confocal microscopy showed co-localization between lysosomal and membrane staining (for CD68 and CD11b respectively), SIM resolved the signals providing a more accurate representation of the cellular biology. The SIM approach used here was fully validated to rule out any image artifacts, a major limitation associated with the steps for superresolution that critically impacts on image quality and data interpretation.

Our study demonstrates that the phagocytic behavior of brain myeloid cells progresses at least until 7 days after the ischemic insult, showing two distinct phases. In the first phase (48 h), lysosomes are clustered close to cell membrane thus indicating active internalization, while in the second phase (7 days) lysosomes are spread over the cytoplasm for final digestion. Lysosomal positioning coordinates cellular metabolic responses to environmental changes [31]. The observed lysosomal membrane clustering at early times after ischemia may indicate an active metabolic state of macrophages [31], whose ability to switch to anaerobiosis ensures survival and uptake functions in the ischemic territory [2]. At later times, lysosome cytoplasmatic engulfment may drive the termination stages of phagocytosis. It has been reported that perinuclear lysosomal clustering influences autophagosome/lysosome fusion rates finally controlling autophagy [31], a critical step to terminate or modify the local immune response [22]. In the context of brain ischemia, myeloid cell autophagy is largely unexplored, with few works reporting its association with increased damage [32]. In line with this, autophagy has been associated with the detrimental M1 polarization of myeloid cells in vitro [33]. The autophagic-like behavior at 7 days after ischemia, reported here, may indicate a mechanism responsible for the M1 polarization, which has been shown to follow the earlier M2 polarization after brain ischemia [1].

The analysis of phagocytosis was based on GLCM applied to SIM images. SIM reaches a theoretical resolution limit of 100 nm, which is quite larger than other methods for superresolved imaging like photoactivated localization microscopy (PALM), stochastic optical reconstruction microscopy (STORM), or stimulated emission depletion (STED), all getting to ≈20 nm [34]. On the contrary, SIM stands as the most versatile and accessible approach and may be used over a wide range of samples with standard fluorescent dyes [35]. Moreover, SIM has a stronger literature on image validation than other superresolution methods [25, 36], which was critical to our purpose. The quality of SIM images can be analyzed by the *SIMcheck* ImageJ toolbox providing image quantitative diagnosis [25]. An image diagnoses toolbox for other superresolution approaches—named *SQUIRREL*—has been only recently introduced [21]. As we envisaged to image brain inflammatory cells that populate a scattered tissue, like the damaged brain cortex after ischemic injury [1], SIM image diagnosis was critical. The specimens analyzed in this study were obtained with the standard protocols that apply to different imaging purposes, including lesion size determination, quantitative immunohistochemistry, and three-dimensional confocal microscopy [1, 15, 26, 37]. Brain specimens were cryostate cut in 20-μm-thick serial sections laid on glasses and stored for any of the aforementioned techniques. Section thickness along with the high scattering and autofluorescence of the ischemic area might hamper satisfactory SIM image quality [38] that needs thorough validation.

The first line of image diagnose is done on raw images, which contain the three grating angles with five phases each, and verifies system calibration, acquisition parameters settings, and sample preparation. Our raw images satisfied three out of four *SIMcheck* outputs, namely CIP, FPJ, and MIV, thus showing uniform illumination through the grating, while failed to provide a good contrast. This is likely due to the characteristics of the ischemic area, which is autofluorescent as dying cells release debris pigment granules like lipofuscin causing autofluorescence, and scatters light. The second line of image diagnosis is done on the reconstructed image in order to check the reconstruction parameters and to give the effective resolution achieved [25]. The reconstructed Fourier plots obtained from the final images in this study showed a radial profile of spatial frequencies with the characteristic ‘flower’ pattern, indicating satisfactory reconstruction. The actual resolution achieved was ≈130 nm when illuminating the lysosomes at λ_exc_ = 561 nm, thus almost doubling the resolution of standard confocal microscopy.

A second validation was done comparing the SIM data with TEM. SIM is indeed a method based on indirect visualization of the structures of interest through labeling with a fluorescent dye. TEM provides images of unlabeled structures therefore offering a solid benchmark of subcellular organization. TEM showed a differential lysosomal distribution at the two time points analyzed which was consistent with the SIM dataset, e.g., clustering at membrane at 48 h and cytoplasmatic spreading at 7 days after ischemia. While TEM still represents the best option for subcellular studies, SIM, proven its reliability, might be preferred since (1) it works on samples which preparation is suitable for other purposes, (2) allows more statistical power, and (3) can be applied to live imaging studies. This latter property is key to assess the dynamics of a given event, providing relevant functional information. As such, we applied time-lapse SIM on live cultured microglia prompted to phagocyte fluorescent beads sized 100 nm by LPS stimulation. The bead signal was analyzed with GLCM and revealed that, as microglia engulfed beads, pixel homogeneity decreased, thus demonstrating that this measure can be used as an index of phagocytosis. We acknowledge that the in vitro setting used here to address phagocytosis has differences than the brain ischemic environment, but this in vitro setting is widely used to study microglia-dependent mechanisms in a pro-inflammatory milieu. We used spherical nanoparticles sized 100 nm that, due to their size and shape, might be quickly internalized by activated microglia through phagocytosis or endocytosis [8, 39–41]. Immunofluorescence done at the final time point on LPS-stimulated microglia showed that lysosomes were in close contact with the beads, thus indicating a phagocytic route of bead uptake. As such, time-lapse SIM on phagocytic microglia culture provided a simplified tool to test and validate the GLCM analysis as phagocytosis proceeds.

Phagocytosis is used by brain myeloid cells in different conditions, such as during brain development to prune synapses and model brain connectivity [42], or responding to altered homeostasis either in protective or detrimental ways [2]. Phagocytosis is mainly studied in vitro [11] or by FACS analysis [43] which requires tissue processing to sort macrophages thus loosing information on cell localization relative to an injured area. A few methods based on microscopy exist to assess phagocytosis in the brain [16, 44]; however, most of them lack enough resolution and perhaps sensitivity. SIM has been successfully applied to analyze engulfed synapses by microglia in the developing or diseased brain on histological preparation [45]. In order to decipher the intracellular changes induced by phagocytosis, SIM was applied on cultured myeloid cells primed to a phagocytic [9] or autophagic [46] behavior. Phagocytosis induced a reorganization of the cytoskeleton characterized by F-actin bundle into fibers preceding engulfment [9]. Autophagy was analyzed by assessing protein localization in the autophagosome [46]. Myeloid cells are highly responsive to brain environmental changes [2], thus a culture system might not reproduce all the stimuli these cells are exposed to after a brain threat [12]. In a recent work, SIM was applied to histological preparations from the human cochlea and provided qualitative data on CD68 expression by macrophages [47].

## Conclusions

Our work is the first providing a quantitative analysis of SIM images describing distinct phases of phagocytosis, namely active internalization vs. final digestion/autophagy, in tissue specimens based on intracellular lysosomal distribution. Image diagnoses ensured SIM data quality ruling out any artifacts. As such, this method can be applied to study phagocytosis in other pathophysiological contexts or in response to treatments targeting inflammation, helping define the functional commitment of myeloid cells. We point out that the present workflow of SIM use and validation should be regarded as the best practice for superresolved imaging, a rapidly evolving field which needs solid protocols to avoid data misinterpretation.

## Additional files

Additional file 1: The ARRIVE Guidelines Checklist. Animal Research: Reporting In Vivo Experiments. (PDF 1066 kb)

Additional file 2: Figure S1 Transmission electron microscopy (TEM) images of neurons in contra- or ipsi-lateral sides to the lesion. Figure S2 DAPI SIM dataset validation by image diagnosis. Figure S3 CD11b SIM dataset validation by image diagnosis. Figure S4 Confocal microscopy and SIM on fixed cells. (DOCX 4503 kb)

## Abbreviations

ARRIVE: Animal Research: Reporting of In Vivo Experiments
ASM: Angular second moment
FFT: Fast Fourier transform
FPJ: Fourier projection
GL: Gray level
GLCM: Gray level co-occurrence matrix
IDM: Inverse difference moment
LAMP: Lysosome-associated membrane glycoprotein
LPS: Lypopolysaccharide
MCA: Middle cerebral artery
MCN: Modulation contrast-to-noise ratio
MIV: Motion and illumination variation
NA: Numerical aperture
PALM: Photoactivated localization microscopy
PFA: Paraformaldehyde
pMCAo: Permanent middle cerebral artery occlusion
ROI: Region of interest
SIM: Structured illumination microscopy
STED: Stimulated emission depletion
STORM: Stochastic optical reconstruction microscopy
TEM: Transmission electron microscopy
