# Supplementary material for: The phagocytic state of brain myeloid cells after ischemia revealed by superresolution structured illumination microscopy
Source: J Neuroinflammation. 2019 Jan 16;16:9. doi: 10.1186/s12974-019-1401-z (PMC6335825; doi:10.1186/s12974-019-1401-z)
Supplement: Supplementary file 2 — Figure S1 Transmission electron microscopy (TEM) images of neurons in contra- or ipsi-lateral sides to the lesion. Figure S2 DAPI SIM dataset validation by image diagnosis. Figure S3 CD11b SIM dataset validation by image diagnosis. Figure S4 Confocal microscopy and SIM on fixed cells. (DOCX 4503 kb) [file 12974_2019_1401_MOESM2_ESM.docx]

**Supplementary information**


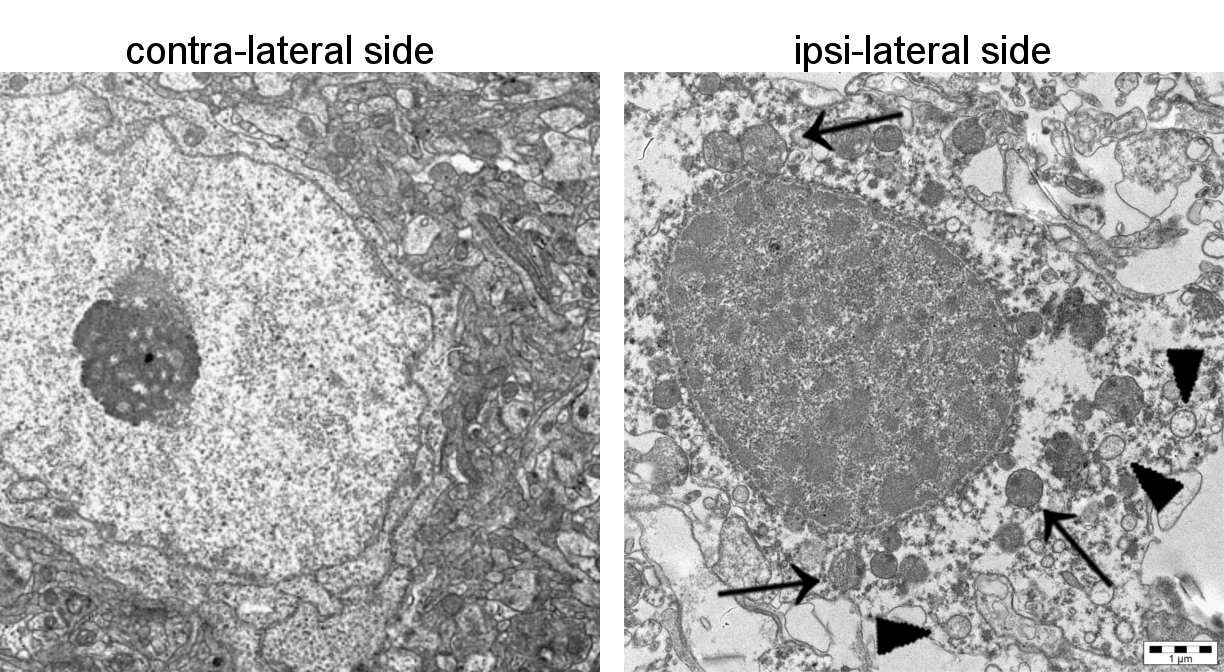


**Supplementary Figure 1. Transmission electron microscopy (TEM) images of neurons in contra- or ipsi-lateral sides to the lesion.** TEM images showing healthy (contra-lateral side, left panel) or damaged (ipsi-lateral side, right panel) tissue. The lesioned area was identified as that showing damaged neuropil and neurons with organelle alterations, such as swelling (arrow) and disruption of cristae (arrowheads) in mitochondria. Scale bar = 1 µm.

**
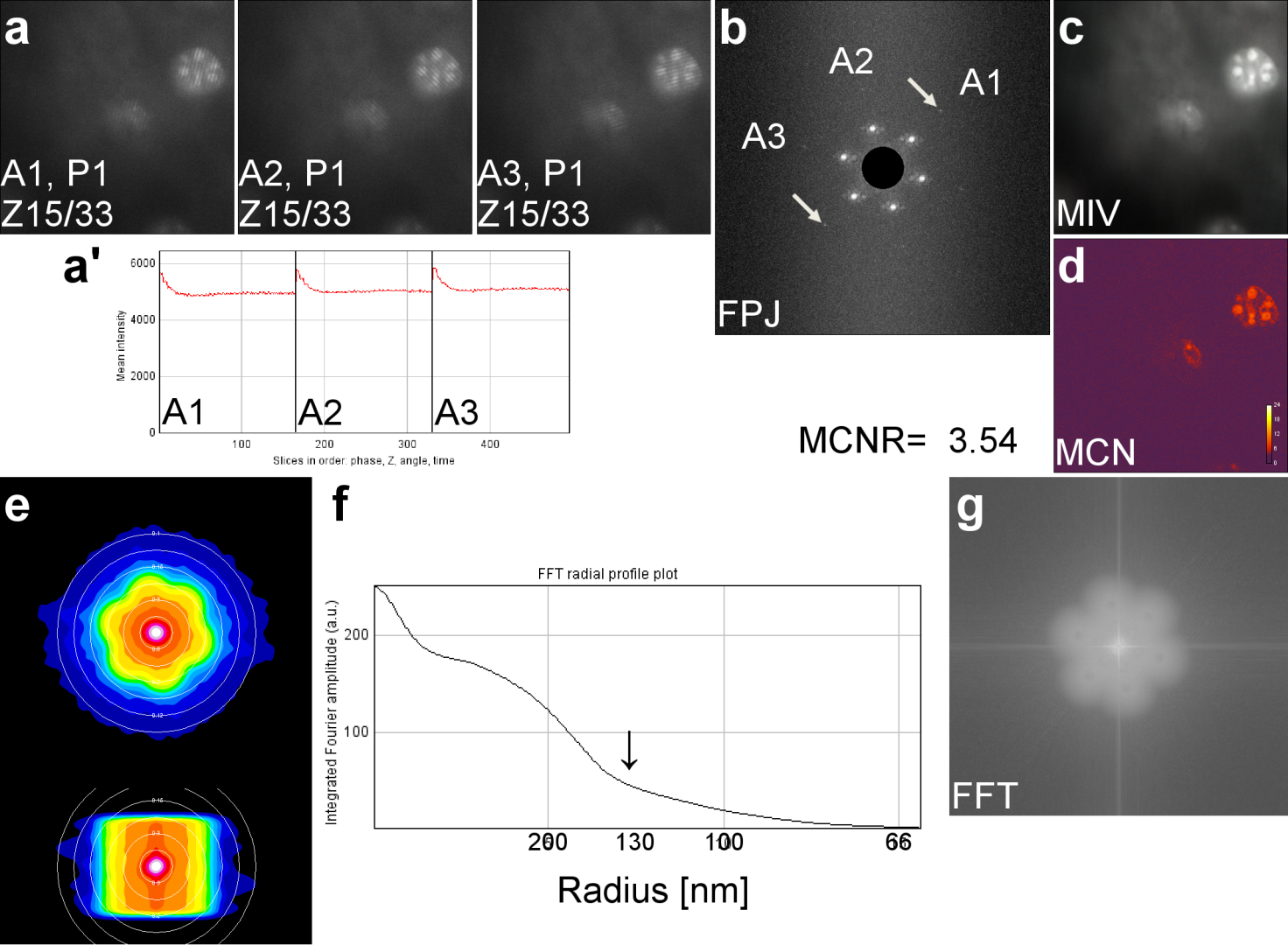
**

**Supplementary Figure 2. DAPI SIM dataset validation by image diagnosis.**

SIMcheck output for 3D-SIM raw dataset of nuclei showing the three illumination angles at the first phase (P1) and at the 15th focal plane (Z15/33, **a**). The channel intensity profile shows limited intensity variation over phases (**a’**). Raw Fourier Projection (FPJ) showing points of high-frequency information for each angle from first (inner spots) to second (outer spots, arrows for angle 1) order stripes (**b**). Motion & Illumination Variation (MIV) based on phase-averaged and intensity-normalized images for each angle showing a gray-white merge output, thus indicating motion stability and evennes of the illumination (**c**). Modulation Contrast-to-noise Ratio (MCN) showing the heatmap of local contrast which is slightly unsatisfactory (<4, **d**). SIMcheck output for 3D-SIM reconstructed dataset of nuclei showing the ‘flower’ pattern in 16-color-coded image for the *xy* plane and the *xz* projection (**e**). The inflection point in the radial profile plot indicates approximate effective resolution achieved in the reconstructed data (≈ 130 nm, arrow, **f**). Fast Fourier Transform (FFT) showing the extended focus image of the ‘flower’ pattern of the reconstructed image (**g**).

**
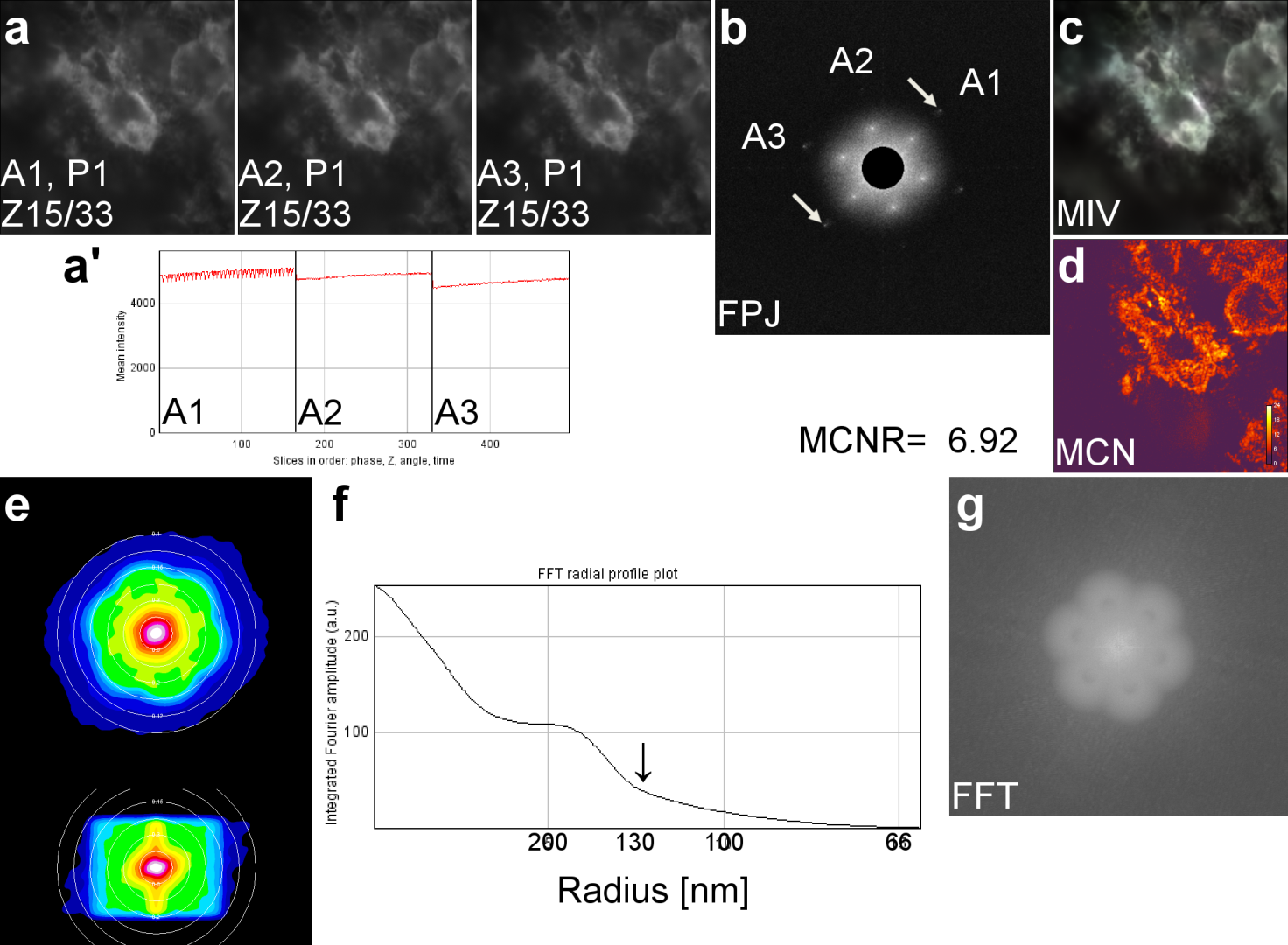
**

**Supplementary Figure 3. CD11b SIM dataset validation by image diagnosis**.

SIMcheck output for 3D-SIM raw dataset of CD11b showing the three illumination angles at the first phase (P1) and at the 15th focal plane (Z15/33, **a**). The channel intensity profile shows limited intensity variation over phases (**a’**). Raw Fourier Projection (FPJ) showing points of high-frequency information for each angle from first (inner spots) to second (outer spots, arrows for angle 1) order stripes (**b**). Motion & Illumination Variation (MIV) based on phase-averaged and intensity-normalized images for each angle showing a gray-white merge output, thus indicating motion stability and evennes of the illumination (**c**). Modulation Contrast-to-noise Ratio (MCN) showing the heatmap of local contrast which is satisfactory (>4, **d**). SIMcheck output for 3D-SIM reconstructed dataset of CD11b showing the ‘flower’ pattern in 16-color-coded image for the *xy* plane and the *xz* projection (**e**). The inflection point in the radial profile plot indicates approximate effective resolution achieved in the reconstructed data (≈ 130 nm, arrow, **f**). Fast Fourier Transform (FFT) showing the extended focus image of the ‘flower’ pattern of the reconstructed image (**g**).


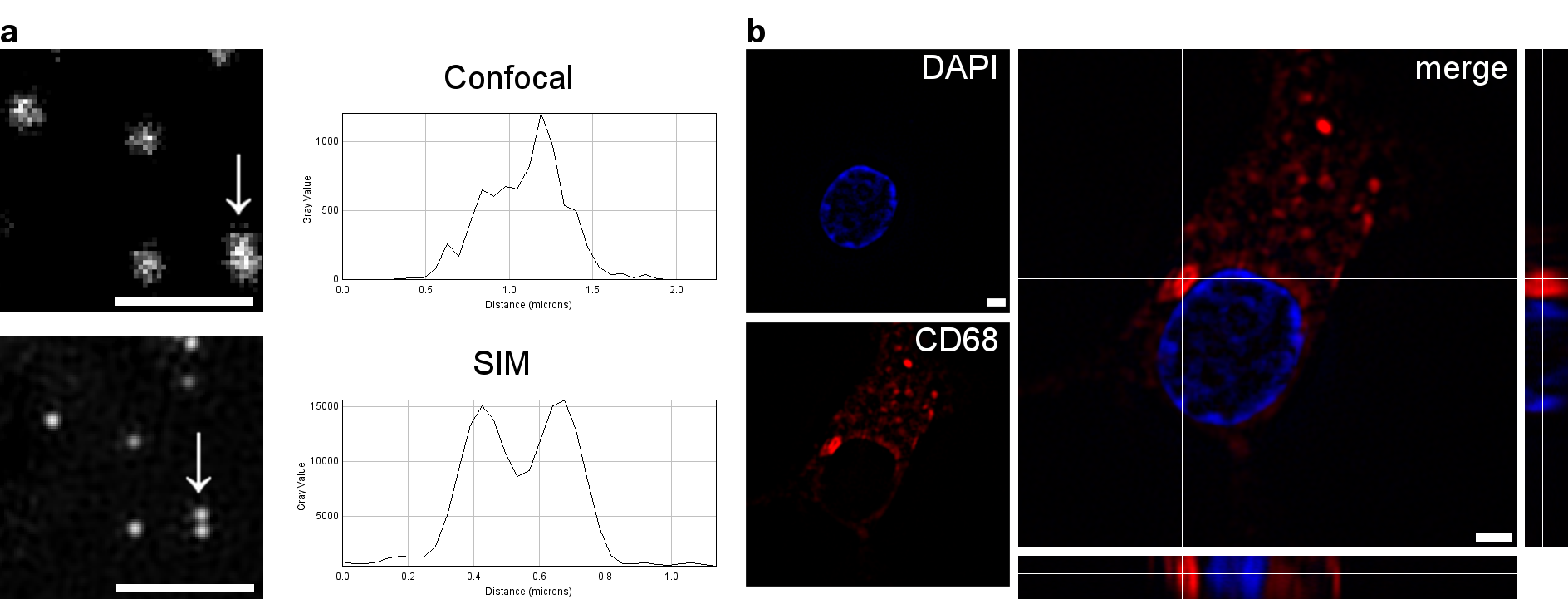


**Supplementary Figure 4. Confocal microscopy and SIM on fixed cells.** At the final time point (170 minutes) cells were fixed for immunofluorescence. SIM using the 60x water immersion objective provided increased resolution than confocal microscopy (**a**, the beads indicated by the arrow was measured in the gray level profile, right panels). Microglia that did not receive beads had few lysosomes in their cytoplasm (**b**). Scale bars = 2 µm.
